# The Use of Kidney Disease Improving Global Outcomes AKI Definitions in AKI Research: COMMENTARY

**DOI:** 10.34067/KID.0000000000000457

**Published:** 2024-07-25

**Authors:** F. Perry Wilson

**Affiliations:** Clinical and Translational Research Accelerator, Department of Medicine, Yale School of Medicine, New Haven, Connecticut

**Keywords:** acute kidney failure, AKI, acute renal failure, creatinine

## Introduction

Although we may not agree how to define it, we all recognize a clinical entity that could be called AKI which is characterized by a change in kidney filtration that occurs over a relatively short period of time. Beyond that point of agreement, the nephrology community continues to debate myriad subpositions: Is a change in filtration truly pathologic? Is a change in filtration truly the *sina qua non* of injury? And of course—whether our current method of assessing filtration—serum creatinine—is adequate to the task of interrogating this highly morbid, costly, and complex issue?

In this issue of *Kidney360*, Drs. McCoy, Chertow, Tamargo, and Parikh take opposing positions on the use of the current Kidney Disease Improving Global Outcomes (KDIGO) AKI Definition in AKI research. Drs. McCoy and Chertow^1^ point out how the ubiquity of serum creatinine measurement facilitates broad scale epidemiologic studies and how the staging system provided by AKI allows for at least some tailoring as to which patients would be eligible for which studies.^2^ Drs. Tamargo and Parikh,^3^ in contrast, suggest that creatinine-based AKI definitions lack nuance and hamper research by lumping diverse AKI etiologies into one epidemiologic entity.

Looking at the landscape of AKI research, both positions seem to be valid. It is undeniably clear that even the small creatinine changes that define stage 1 AKI are strongly associated with adverse outcomes.^4,5^ However, this does not necessarily mean that the decrease in filtration is causally linked to those outcomes. AKI's requirement for a change in creatinine creates some potential biases as we have described before. For example, sicker individuals will have more creatinine concentrations measured during a hospitalization, and more frequent measurement will increase the chance of false-positive AKI.^6^ Nevertheless, studies that have attempted to account for various confounders continue to show an independent association between small creatinine changes and adverse outcomes including death.^7,8^

On the other hand, AKI research has made little progress in *changing* outcomes for patients with AKI, at least when the KDIGO definition is used. For example, we enrolled 6030 patients with KDIGO-defined stage 1 AKI in a randomized trial of AKI alerts. There was no difference between the intervention and control groups in the primary outcome (a composite of progression of AKI, dialysis, or death).^9^ I am tempted to blame the overly sensitive, overly broad AKI definition as the reason for the negative results. Of course, I am biased in that interpretation—after all, it is easier to blame the AKI definition than the design of our own intervention. The broad failure of multiple AKI intervention trials may suggest, simply, that we need better interventions.

Both authors acknowledge that creatinine's value stems, at least in part, from its ubiquity in clinical medicine. The basic metabolic panel or Chem-7, containing creatinine, can be traced to the invention of the autoanalyzer in 1957 and has remained essentially unchanged for more than 5 decades.^10^ While I can imagine a world of daily Neutrophil Gelatinase-Associated Lipocalin or KIM1 or CXCL9 levels—the costs associated with that level of testing (both in terms of dollars and false positives) would make that world painful to live in. Indeed, even gold standard biomarkers like troponin require clinical suspicion before they are ordered. And how are we to be clinically suspicious that kidney function has declined without observing a change in creatinine?

I am inclined to, if not embrace, at least become reconciled to our creatinine-dominated AKI world. However, the current KDIGO definition, which includes both an absolute change and a percent change in creatinine, creates some real difficulties. Under the current paradigm, an individual with a baseline creatinine of 3.0 which then rises to 3.3 (a 10% change) within 48 hours would have AKI. It is clear that the current definition, which is admittedly sensitive, is *more sensitive* among those with CKD. This may have the effect of biasing even those retrospective epidemiologic analyses for which KDIGO criteria have been so valuable.

There are, of course, other ways to interrogate creatinine to define AKI. We could embrace an entirely percent-based definition (I would suggest that a change of 25% in 48 hours of 50% in 7 days nicely threads the needle). More advanced calculations that embrace the biologic and laboratory variability of creatinine have also been proposed.^11^ However, evaluating these definitions against each other can be difficult—as is often the case when information is limited we may simply be trading sensitivity for specificity or *vice versa* as we try different definitional paradigms.

In sum, I agree that, for now, creatinine and even the current KDIGO method of interrogating creatinine change are appropriate tools to diagnose AKI in hospitalized patients. I also agree that we should recognize these as screening tests which should, at least in prospective studies, prompt further evaluation (potentially with relevant biomarkers) to further investigate the etiology of AKI and to help develop more specific therapies. Finally, it may be reasonable for KDIGO to consider adding a biomarker-based definition of AKI that is independent of creatinine to encourage further clinical adoption and research into this critical area of nephrology.
